# Restoration of microRNA function impairs MYC-dependent maintenance of MLL leukemia

**DOI:** 10.1038/s41375-020-0768-2

**Published:** 2020-02-24

**Authors:** Shouhai Zhu, Xiaoyan Cheng, Ruiheng Wang, Yuting Tan, Maolin Ge, Dan Li, Qiongyu Xu, Yan Sun, Chunjun Zhao, Saijuan Chen, Han Liu

**Affiliations:** State Key Laboratory of Medical Genomics, Shanghai Institute of Hematology, Rui Jin Hospital, School of Medicine and School of Life Sciences and Biotechnology, Shanghai Jiao Tong University, 200025 Shanghai, China

**Keywords:** Leukaemia, Oncogenesis

## To the Editor:

MicroRNAs (miRNAs) are a class of small noncoding RNAs that have critical functions in gene silencing by binding to complementary mRNAs to induce their degradation or translational repression [1]. Interestingly, only a small fraction of miRNAs can exert their function in the form of miRNA-induced silencing complex (miRISC) [2]. Unfortunately, although dysregulated expression or processing of cancer-related miRNAs has been demonstrated to play a crucial role in oncogenesis, the contribution of miRNA dysfunction is still poorly understood.

MLL-rearranged leukemias generally have a poor prognosis and account for 10% of overall acute lymphoblastic leukemia (ALL) and acute myeloid leukemia (AML) cases [3]. MLL translocations encoding chimeric fusion proteins comprising the N-terminus of MLL in frame with various fusion partner proteins are characteristically found in MLL leukemias [3]. MLL is a histone H3 Lysine4 (H3K4) methyltransferase and is proteolytically cleaved into two distinct subunits, MLL^N320^ and MLL^C180^, which noncovalently interact to form an intramolecular complex involved in epigenetic transcriptional regulation. Interestingly enough, we recently uncovered an unexpected role for MLL protein in miRNA-mediated translational repression [4]. Our studies showed that the MLL^C180^ subunit alone could colocalize with miRISC components in cytoplasmic processing bodies (P-bodies), and affect the function of a subset of miRNAs such as the *let-7* family [4].

Multiple miRNAs have been found to be dysregulated in MLL leukemias [5, 6]. However, since only a small fraction of miRNAs are functional, it is not clear whether the expression levels of miRNAs reflect their actual contribution to the pathogenesis of MLL leukemia. Considering that the abundance of wild-type MLL protein was reduced in MLL leukemic cells [7, 8], we reasoned that the function of a subset of miRNAs would be compromised and may play a critical role in the pathogenesis of MLL leukemia.

**Fig. 1:**
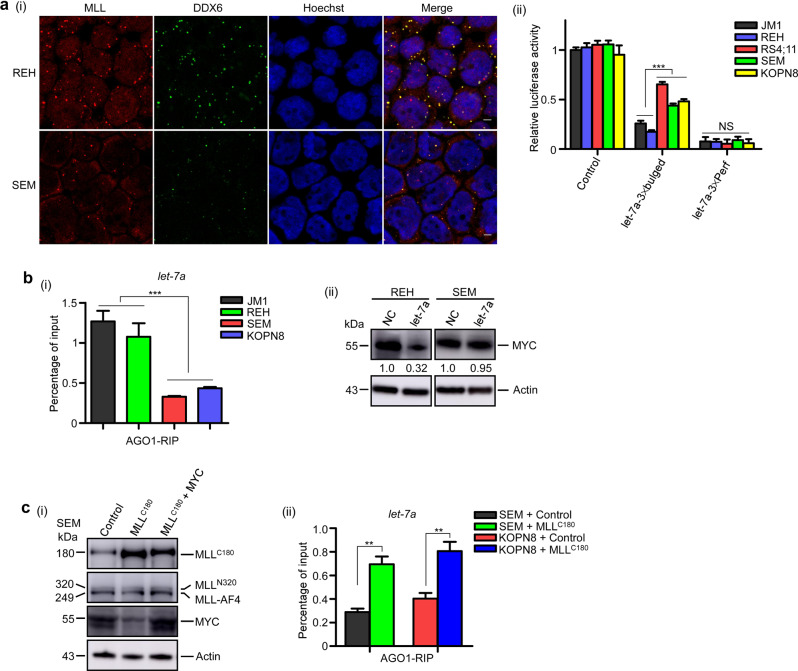
MLL-fusion leukemic cells showed an impaired miRNA-mediated translational repression. **a** (i) REH and SEM cell lines were probed with antibodies to DDX6 for immunofluorescence assay. REH cell line harbors wild-type *MLL* gene and SEM cell line harbors *MLL-AF4* gene. Scale bar, 5 μm. (ii) The effects of MLL translocations on the function of endogenous *let-7a* were analyzed using dual luciferase reporter assays. The reporter activity was normalized to JM1 cells transfected with empty reporter vector. **b** (i) Extracts of JM1, REH, SEM, and KOPN8 cells were subjected to anti-AGO1 RIP assays. The pull-downed RNAs were analyzed by qRT-PCR using primers for *let-7a*. (ii) REH and SEM cells were transfected with NC and let-7a. Proteins were detected by western blot with anti-MYC antibody at 24 h post transfection. **c** (i) SEM cells transduced with *MLL*^*C180*^, together with or without *MYC* were subjected to western blot assays. Antibodies were used as indicated. MLL-AF4 fusion proteins were detected using antibody specifically recognizing the amino terminus of MLL. (ii) SEM and KOPN8 cells transduced with or without *MLL*^*C180*^ were subjected to anti-AGO1 RIP assays. Pull-down RNAs were analyzed by qRT-PCR using primers for *let-7a*. *P < 0.05, **P < 0.01, ***P < 0.001. Data represent mean and s.e.m. of three independent experiments.

We first addressed how P-body formation and miRNA-mediated gene silencing were affected in MLL leukemic cells. Immunofluorescence results showed that the number of DDX6- and DCP1A-marked P-bodies in MLL leukemic cells, including RS4;11, SEM, KOPN8, and THP-1 cells, was significantly fewer than in non-MLL leukemic lines such as JM1, REH, and U937 cells (Fig. 1a (i), and Supplementary Fig. S1a–c). Furthermore, the capacity of ectopically expressed *let-7a* or *CXCR4* to silence their bulged miRNA reporters, but not perfect siRNA reporters, was markedly reduced in MLL leukemic cells (Fig. 1a (ii) and Supplementary Fig. S2a, b). These results were consistent with our previous findings that MLL was required for miRNA-mediated translational repression of partially matched mRNAs, but not for cleavage of perfectly matched mRNAs [4]. These defects in the MLL leukemic cells were associated with a reduced level of MLL^C180^ but not P-body proteins (Supplementary Fig. S2c). Further introduction of MLL^C180^ restored miRNA-mediated gene silencing in MLL leukemic cells (Supplementary Fig. S2d), indicating that the impairment in miRNA-mediated gene silencing in MLL leukemic cells was caused by downregulating wild-type MLL, especially the MLL^C180^ subunit.

*MYC* represents a critical cooperation pathway and a therapeutic target in MLL-rearranged AML that is frequently upregulated in this disease [9, 10]. We further showed that the proliferation of MLL-rearranged B-ALL cells was decreased upon *MYC* depletion (Supplementary Fig. S3a, b) and that MYC protein abundance in MLL-rearranged B-ALL cells was much higher than in non-MLL-rearranged B-ALL cells (Supplementary Fig. S3c), implying that both AML- and B-ALL-type MLL leukemic cells are generally dependent on high levels of MYC protein. However, although *MYC* is regarded as a downstream target of MLL-fusion proteins [9], the expression level of *MYC* mRNA was not proportionally increased (Supplementary Fig. S3c), which was also validated using publicly available microarray datasets of well-characterized primary ALL and AML patient samples (Supplementary Fig. S3d), suggesting that translational repression of *MYC* mRNA was reduced in MLL leukemic cells.

Since our previous study demonstrated that MLL was required for *let-7a*-mediated translational repression, and *MYC* is one of the most well-established *let-7a* targets, we reasoned that the inability of endogenous *let-7a* to repress translation of its target mRNAs may partly contribute to the high expression level of MYC protein in MLL leukemic cells. Therefore, we determined whether the translational repression function of endogenous *let-7a* was impaired in MLL leukemic cells. Since translational suppression of mRNA targets by mature miRNAs preferentially requires AGO1 [11], we performed AGO1 RNA immunoprecipitation (RIP) experiments and showed that the binding of both *let-7a* and *MYC* mRNA to AGO1 were reduced in MLL leukemic cells (Fig. 1b (i), and Supplementary Fig. S4a (i–ii), b). The pull-down assay using biotinylated *let-7a* further validated that the binding of AGO1 to *let-7a* was reduced in MLL leukemic cells (Supplementary Fig. S4c). Moreover, the protein levels of MYC decreased significantly after *let-7a* transfection in non-MLL-rearranged REH and JM1 cells, but not in MLL-rearranged SEM, RS4;11, and KOPN8 cells (Fig. 1b (ii) and Supplementary Fig. S4d, e). These results suggested that the expression of MYC proteins could escape translational repression by *let-7a* in MLL leukemic cells.

To confirm that the impaired translational repression of *MYC* by *let-7a* and the high level of MYC in MLL leukemic cells were caused by reduced MLL^C180^ expression, we evaluated how restoring MLL^C180^ affected miRNA function and MYC expression. The introduction of MLL^C180^ in SEM and KOPN8 cells decreased MYC expression and impaired cell proliferation, which could be recovered by MYC overexpression (Fig. 1c (i) and Supplementary Fig. S5a, b). However, the expression level of MLL-AF4 (Fig. 1c (i)) and MLL-ENL (Supplementary Fig. S5a) proteins was similar between the control and MLL-C-rescued MLL leukemic cell lines, suggesting the decreased MYC protein level in the MLL-C-rescued cells was not associated with a lower level of MLL-fusion proteins. In addition, immunofluorescence results showed that introduction of MLL^C180^ increased the number of P-bodies (Supplementary Fig. S5c). The binding of *let-7a* and *MYC* mRNA to AGO1 in SEM and KOPN8 cells after MLL^C180^ introduction was also enhanced as revealed by RIP assays (Fig. 1c (ii) and Supplementary Fig. S5d, e). These data indicated that the reduction of MLL^C180^ played a causal role in the miRNA functional deficiency in MLL leukemic cells.

LIN28A and LIN28B can be transcriptionally activated by MYC [9], and in turn enhance the expression of *MYC* by blocking the expression of *let-7a* [12], thus forming a regulatory circuit. Although the introduction of MLL^C180^ could significantly downregulate the MYC expression, it had little effect on the levels of LIN28A/LIN28B and mature *let-7a* (Supplementary Fig. S6a, b). Furthermore, depletion of endogenous *LIN28A/LIN28B* showed little effects on the binding of *let-7a* and *MYC* mRNA to AGO1 (Supplementary Fig. S6c (i–ii), d (i–ii)). These results ruled out the contribution of LIN28 in the context of MLL^C180^ regulated *let-7a* dysfunction, highlighting the dominant role of MLL^C180^ in controlling *let-7a*-mediated *MYC* suppression.

**Fig. 2:**
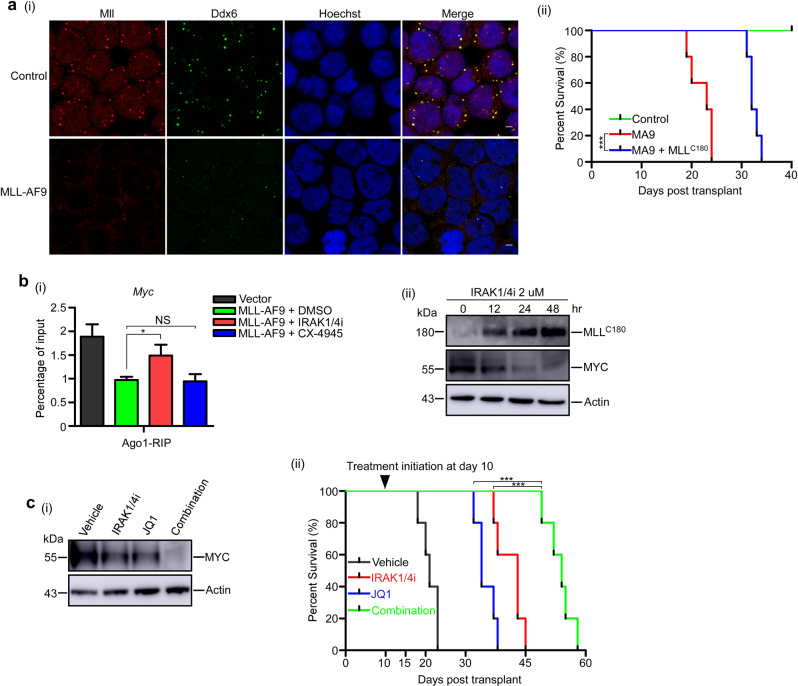
Impaired miRNA-mediated translational repression in MLL-fusion leukemic cells contributes to MYC-dependent survival. **a** (i) Mouse bone marrow progenitor cells transduced with control or *MLL-AF9* gene were probed with antibodies to Ddx6 for immunofluorescence assay. Scale bar, 5 μm. (ii) Effect of *MLL*^*C180*^ on *MLL-AF9* mediated leukemogenesis in vivo. Kaplan–Meier survival curves were shown for three groups of normal and transplanted mice including *MLL-AF9* and *MLL-AF9* + *MLL*^*C180*^ in a secondary bone marrow transplantation Assay (*n* = 5 per each group). **b** (i) Mouse bone marrow cells transduced with empty vector (control) or *MLL-AF9*, were treated with DMSO (control), IRAK1/4 inhibitor (2 μM), or CX-4945 (2 μM) for 48 h. Then cells were collected for the anti-Ago1 RIP assays. Pull-down RNAs were analyzed by qRT-PCR using primers for *Myc*. (ii) Western blot analyses of the time-dependent effects of IRAK1/4 inhibitor treatment on MLL^C180^ and MYC expression in *MLL-AF9* primary leukemia cells. Actin was used as a loading control. **c** (i) *MLL-AF9* primary leukemia cells were treated with vehicle control, IRAK1/4 inhibitor (2 μM), JQ1 (100 nM), or combination for 24 h. Cell lysates were collected and analyzed by western blot for the expression of MYC. (ii) Kaplan–Meier survival curves of secondary transplanted NOD-SCID mice after vehicle, JQ1, IRAK1/4 inhibitor, or combination treatment at day 10 (*n* = 5 per each group). IRAK1/4 inhibitor (8 mg/kg), JQ1 (50 mg/kg), or combination were administered every other day by intraperitoneal injection for a total of five treatments. *P < 0.05, **P < 0.01, ***P < 0.001. Data represent mean and s.e.m. of three independent experiments.

To further strengthen our findings, we examined whether miRNA-mediated gene silencing was impaired in primary MLL leukemic cells. Compared with the control cells, the *MLL-AF9*-transduced primary mouse bone marrow progenitor cells showed a marked reduction in the number of P-bodies (Fig. 2a (i)). In addition, the capacity for silencing the miRNA reporters (Supplementary Fig. S7a, b) and the binding of *let-7a* and *MYC* mRNA to AGO1 (Supplementary Fig. S7c) were also significantly reduced. These defects were correlated with a high expression level of MYC protein in primary MLL leukemic cells (Supplementary Fig. S7d, e). Furthermore, ectopically expressed MLL^C180^ could partially rescue *let-7a*-mediated gene silencing in *MLL-AF9*-transduced primary cells (Supplementary Fig. S7a, c) and significantly delay the development of leukemia in the xenografted mice (Fig. 2a (ii) and Supplementary Fig. S7f).

A previous report revealed that pharmacologically inhibiting the IRAK pathway could substantially improve survival of mice with MLL leukemia by stabilizing the wild-type MLL protein [13]. We hypothesized that IRAK inhibition could rescue the function of *let-7a* and decrease the expression of MYC via restoration of MLL protein levels. We first examined the effect of an IRAK1/4 inhibitor (IRAK1/4i) on miRNA-mediated gene silencing in *MLL-AF9*-transduced primary cells. We observed that the capacity of *let-7a* for silencing the miRNA reporters (Supplementary Fig. S8a) and the binding of *let-7a* and *MYC* mRNA to AGO1 (Fig. 2b (i) and Supplementary Fig. S8b) were significantly increased upon IRAK1/4i treatment. In contrast, casein kinase II (CKII) inhibitor CX-4945, which increases the level of full-length MLL protein by blocking taspase1-dependent MLL processing [14], had very little effect in these assays. Furthermore, treatment with IRAK1/4i, but not CX-4945, led to significant MLL^C180^ induction and MYC reduction (Fig. 2b (ii) and Supplementary Fig. S8c, d). These results further support our previous finding that only free MLL^C180^ was implicated in miRNA-mediated translational suppression [4].

*MYC* represents a critical therapeutic target in MLL-rearranged leukemias [10]. Bromodomain and extra-terminal (BET) protein inhibitors have shown profound efficacy against MLL-rearranged leukemia by inhibiting several key targets including MYC [10]. In fact, MYC restoration constitutes one of the major mechanisms of BET inhibitor resistance [15]. We therefore reasoned that IRAK1/4i may improve the efficacy of BET inhibitors by synergistically downregulating MYC in MLL leukemias. Indeed, in line with this hypothesis, concomitant IRAK1/4 and BET inhibition synergistically led to a significant reduction of MYC protein in MLL-AF9 primary leukemia cells (Fig. 2c (i)), and synergistically inhibited the development of MLL-rearranged leukemia in a murine xenograft model (Fig. 2c (ii) and Supplementary Fig. S8e), suggesting combination therapy with IRAK1/4 and BET inhibitors can achieve better efficacy against MLL leukemias.

In summary, our results demonstrated that the dysfunction of *let-7a* caused by a reduced level of MLL was essential for maintaining MYC protein at a high level and sustaining the survival of MLL leukemic cells. Thus, our work has uncovered a functional link between miRNA dysfunction and MLL-rearranged leukemia. Moreover, our work revealed that IRAK1/4 inhibition can improve the efficacy of BET inhibitors in MLL leukemias by restoring the protein level of MLL and the MYC-suppressing function of *let-7a*. We thus propose a novel rationale for synergistically targeting MYC with both IRAK and BET inhibitors as part of a comprehensive therapeutic approach for treatment of MLL leukemias.

## Supplementary information

Supplementary Information
