# Supplementary material for: Restoration of microRNA function impairs MYC-dependent maintenance of MLL leukemia
Source: Leukemia. 2020 Feb 24;34(9):2484–8. doi: 10.1038/s41375-020-0768-2 (PMC7449869; doi:10.1038/s41375-020-0768-2)
Supplement: Supplementary file 1 — Supplementary Information [file 41375_2020_768_MOESM1_ESM.docx]

**Supplementary Information**

**Materials and methods**

**Construction of plasmids**

To generate pmirGLO dual luciferase reporters whose expression is controlled by let-7a, annealed oligonucleotides were inserted into pmirGLO miRNA target expression vector (Promega) to generate reporters bearing perfect (CAACTATACAACCTACTACCTCAAACTATACAACCTACTACCTCAAACTATACAACCTACTACCTCAT; pmirGLO-3xperfect) and bulged (CGCACAGCCTATTGAACTACCTCACTCGGAGCACAGCCTATTGAACTACCTCAGGCCTGCACAGCCTATTGAACTACCTCAT; pmirGLO-3xbulged) let-7a target sites in the 3’UTR region. To construct shRNA vectors for *MYC*, *LIN28A* and *LIN28B*, hairpin-encoding oligonucleotides against *MYC* (GATGAGGAAGAAATCGATG; CCTGAGACAGATCAGCAACAA), *LIN28A* (CCTGGTGGAGTATTCTGTATT; TGCTACAACTGTGGAGGTCTA) and *LIN28B* (GCAGGCATAATAAGCAAGTTA; GCCTTGAGTCAATACGGGTAA) were annealed and ligated into pLKO.1 vector. Vectors expressing full-length MLL, MLL^N320^ or MLL^C180^ have been described previously [1]. Full length *MYC* was cloned from 293T cDNA and inserted into the pCDH vector (System Biosciences). The primer sets were:

*MYC*:

sense: CTGGATTTTTTTCGGGTAGTGG;

antisense: CGCACAAGAGTTCCGTAGCTG.

**Cell culture and transfection**

JM1, REH, RS4;11, SEM, KOPN8, U937 and THP-1 leukemia cell lines were maintained in RPMI-1640 medium supplemented with 10% FBS and 1% 100×Pen Strep. All cells have been described previously [1]. All cell culture reagents were obtained from Life technologies. For IRAK1/4 inhibitor (Selleck) and Casein kinase II (CKII) inhibitor (TargetMol) treatments, the indicated concentration (2 μM) was added to the cell culture for the indicated times before harvesting the cells for the Western blot analysis, luciferase reporter assay or RNA immunoprecipitation (RIP) experiment. For the source of cell lines used in this study, JM1 cell line was obtained from American Type Culture Collection (ATCC); REH, RS4;11, SEM, KOPN8, U937 and THP-1 cell lines were obtained from DSMZ. All cell lines were routinely tested for mycoplasma contamination.

For miRNA transfection, Cy3 labeled Agomir-let-7a-5p mimic (Sangon) or Agomir-negative control (NC) were transfected into recipient cells with the Xfect RNA Transfection Reagent (Clontech). For the RIP experiments, 0.3 μg of let-7a mimic were used for the transfection. After 24 hours, the transfection efficiencies were validated by either qRT-PCR or Western blot analysis. For plasmids transfection, FuGENE HD Transfection Reagent (Promega) was used according to the protocol.

**Flow-cytometry analysis**

For the let-7a transfection experiments, flow cytometric analyses were conducted to evaluate the transfection efficiency. Briefly, cells were harvested at 24 hours post-transfection followed by 2 times washes using PBS solution and the flow-cytometric data were acquired using the LSRFortessa^TM^ X-20 flow cytometer (Becton Dickinson). Then data were analyzed with the FlowJo software (TressStar).

**Antibodies and Western blot**

The antibodies used in this study were: anti-MYC (Santa Cruz; sc-40), anti-DCP1A (for Western blot: Sigma, D5444; for IF: Sigma, WH0055802M6), anti-DDX6 (for Western blot: GeneTex, GTX102795; for IF: Santa Cruz, sc-376433), anti-EDC3 (Santa Cruz, sc-365024), anti-EDC4 (Santa Cruz, sc-137444), anti-MLL1 (Cell Signaling, 14689, recognizing amino-terminal of MLL), anti-AGO1 (Abcam, ab5070), anti-LIN28A (Cell Signaling, 3978), anti-LIN28B (Cell Signaling, 4196), anti-Tubulin (Cell Signaling, 2128), anti-Actin (Sigma, A1978), anti-GAPDH (Sigma, G8795) and MLL-CT antibody that recognizes MLL^C180^ (aa2829-2883) has been described previously [1]. Unless specified, MLL-CT was used for Western blot or IF. All the antibodies were used according to the manufacturer’s recommendations. Antibodies were detected using the enhanced chemiluminescence method (Western Lightning, PerkinElmer) and immunoblot signals were acquired with the AI600 Imaging system (GE).

**Immunofluorescence (IF)**

Immunofluorescence experiments were performed as described previously [2]. Briefly, cells were fixed with 4% paraformaldehyde in PBS for 30 min at room temperature and permeabilized in 0.2% (v/v) Triton X-100 in PBS for 20 min at room temperature. The nonspecific binding was blocked for 60 min in PBS containing 1% BSA and 0.1% Tween-20 and probed in the same buffer with primary antibodies overnight at 4 ℃. The slides were washed 3 times in PBS containing 0.1% Tween-20 and were incubated with the Alexa-labelled secondary antibodies (Thermo Fisher) and Hoechst33342 (Thermo Fisher) for 1 hour. The coverslips were mounted in Fluoromount Aqueous Mounting Medium (Sigma) and imaged using TCS SP8 confocal microscopes (Leica). For z-stack analysis, optical sections were obtained along with the z axis at 0.5 μm intervals. Images were analyzed using the LAS AF Lite and Image J software.

**RNA isolation and qRT-PCR**

miRNA was isolated using *mir*Vana miRNA Isolation Kit (Life Technologies) and dissolved in the appropriate amount of RNase-free water as indicated. mRNA was extracted according to the manufacturer’s instructions (Sangon). RNAs concentration was measured by QuantiFluor RNA System (Promega). Reverse transcription was performed using GoScript Reverse Transcription System (Promega). qPCR was performed with Power SYBR Green master mix (Applied Biosystems) in triplicate using the following primer sets:

*hsa-let-7a-5p*

sense: AAGGCGGTGAGGTAGTAGGTTGT;

antisense: ATCCAGTGCAGGGTCCGAGG;

reverse transcription primer: GTCGTATCCAGTGCAGGGTCCGAGGTATTCGCACTGGATACGACAACTAT.

*MYC*

sense: AATGAAAAGGCCCCCAAGGTAGTTATCC;

antisense: GTCGTTTCCGCAACAAGTCCTCTTC.

*GAPDH*

sense: CATGTTCGTCATGGGGTGAACCA;

antisense: AGTGATGGCATGGACTGTGGTCAT.

*RNU6B*

sense: CTCGCTTCGGCAGCACA

antisense: AACGCTTCACGAATTTGCGT

Mouse *Myc*

sense: TAACTCGAGGAGGAGCTGGA

antisense: GCCAAGGTTGTGAGGTTAGG

Mouse *Gapdh*

sense: TGCACCACCAACTGCTTAG

antisense: GGATGCAGGGATGATGTTT

**Luciferase reporter assay**

Cells were transfected with dual luciferase reporters containing three matched or mismatched *let-7a* binding sites and Agomir-negative control (NC) or Agomir-let-7a-5p mimic (let-7a). At 24 h post-transfection, cells were harvested and dual luciferase reporter assays (Promega) were performed according to manufacturer’s protocol and quantified with a GloMax 20/20 Luminometer (Promega). The *CXCR4* reporter assays were performed as described previously [3].

**Cell proliferation assay**

MTS (Promega) was used to measure the effects of *MYC* or *MLL^C180^* on the proliferation of SEM and KOPN8 cell lines. Briefly, about 5×10^3^ cells per well were seeded into 96 well plates in triplicate and cultured for indicated time. MTS solution was added to the well and incubated for 4 hours. Then absorbance at 490 nm and proliferation curves were measured.

**RIP**

RNA immunoprecipitation (RIP) experiment was performed as described previously [2]. Briefly, 10 million cells were collected, pelleted by centrifuge for 5 min at 1,000 g and washed once with cold PBS (1 ml). The cell pellets were re-suspended with 500 μl lysis buffer (140 mM NaCl, 50 mM Tris-HCl pH 8.0, 0.5% Triton X-100) with protease inhibitor cocktail (Roche) and 400U/ml RNase inhibitor (Promega). Then the mRNP lysate was incubated on ice for 10 min and centrifuged at 12,000 rpm for 10 min. Cell lysate was incubated with indicated antibodies and Dynabeads protein G (Life technologies); the tube was rotated continuously at 4 ℃ overnight. The beads were collected, washed 4 times with 1 ml ice-cold lysis buffer with RNase inhibitor. Then total RNAs were isolated for qRT-PCR or RNA-sequencing. For RIP-seq, isolated RNAs were sequenced using HiSeq2000 (Illumina).

**RNA Pull-down**

REH and SEM cells were transfected with the control miRNA or biotin-labeled let-7a at a final concentration of 100 nM, Xfect RNA Transfection Reagent (Clontech) was used as a transfection reagent. To prepare cell lysates, REH and SEM cells were harvested into 500 μl cold lysis buffer. The lysates were centrifuged at 12000 rpm for 10 min, and the supernatant was used for the Pull-down assay. Biotin-labeled let-7a-5p or scrambled miRNAs, synthesized by Sangon, were incubated with REH or SEM cellular lysates for 12 h at 4 ℃. Then Streptavidin agarose beads (Life technologies) were added to retrieve the protein immunoprecipitates. The beads were collected, washed 3 times with 1 ml ice-cold lysis buffer and bound proteins were eluted by the addition of 1× LDS sample buffer (Life technologies). let-7a-immunoprecipitated proteins were subjected to the Western blot assays.

**Lentivirus Production and Infection**

Lentivirus for sh*MYC*, sh*LIN28A*, sh*LIN28B*, pCDH-*MYC* and pCDH-*MLL^C180^* as well as their controls were packaged with pMD2.G and pSPAX2. Briefly, 3 μg pMD2.G, 9 μg pSPAX2 and 12 μg lentivirus plasmids were co-transfected into 293T cells in 10 cm cell culture dish using ProFection Mammalian Transfection System (Promega). The lentivirus particles were harvested at 48 hours after transfection and concentrated. For infection, the concentrated lentivirus particles were added into the medium with 8 ug/ml polybrene and cultured at 37°C for 24-48 hours before they were washed out.

**Mouse Studies**

Normal mouse bone marrow (BM) progenitor cells of C57BL/6 (CD45.2) mice were retrovirally transduced with MSCVneo (as control), MSCVneo-MLL-AF9 (*MLL-AF9*), together with or without *MLL^C180^* (*MLL-AF9* + *MLL^C180^*), through two rounds of spinoculation, to get the primary leukemia cells. Then, retrovirally transduced donor cells were injected by tail vein into irradiated 6 to 8 weeks old NOD-SCID recipient mice with 2x10^6^ donor cells along with 2x10^5^ BM support cells per recipient mouse. For secondary transplantation, leukemic BM cells isolated from the primary recipient mice were further transplanted into lethally irradiated 6 to 8 weeks old NOD-SCID secondary recipient mice *via* tail vein injection, with the same dosage as described above. For animal treatment, 10 days after *MLL-AF9* primary leukemia cell transplantation, mice were randomized for four groups (*n* = 5 per each group) and treated with 8 mg/kg IRAK1/4 inhibitor, 50 mg/kg JQ1 or combination, or similar volume of vehicle (5% DMSO and 95% PBS) by intraperitoneal injection every other day for 10 days. Mice were monitored for leukemia development and leukemia was confirmed after the mice were sacrificed upon signs of illness (partial paralysis, weight loss, malaise, hunched back, and palpable spleen). JQ1 compound was purchased from Selleck. All laboratory mice including male or female were maintained in the animal facility at the Shanghai Jiao Tong University. All protocols on mice in our experiments were approved by Institutional Animal Care and Use Committee of the Shanghai Jiao Tong University.

**Microarray data analysis**

Raw microarray datasets of primary acute myeloid leukemia (AML) and acute lymphoblastic leukemia (ALL) patient samples were downloaded from Gene Expression Omnibus (GEO). Data were collected from at least three different groups and all of them were generated using the GPL570 Affymetrix Human Genome U133 Plus 2.0 Array platform. The raw AML datasets use in this study were accessible under GSE14062, GSE19577, GSE35784, GSE17855 and GSE52891. The raw ALL datasets used were accessible under GSE14834, GSE13351, GSE19475, GSE58565 and GSE11877. A few T-ALL patient samples in ALL datasets were filtered out. In each group, the profiles were merged before confirming log2 transformation followed by normalization using the R package ‘limma’ to remove difference between arrays. We selected the probe ‘202431_s_at’ with a stronger signal as *MYC* and compared the *MYC* expression between *MLL* germline and rearranged leukemia. Wilcoxon rank-sum tests were performed for the pairwise comparisons.

**Statistical analysis**

Data are displayed as the mean ± s.e.m., and *P*-values were calculated using two-tailed Student *t*-test or log-rank test for significance. The statistically significance level is indicated as * for *P*<0.05, ** for *P*<0.01, or *** for *P*<0.001.

**References:**

1. Liu, H., Cheng, E.H. & Hsieh, J.J. Bimodal degradation of MLL by SCF^Skp2^ and APC^Cdc20^ assures cell cycle execution: a critical regulatory circuit lost in leukemogenic MLL fusions. Genes Dev. 2007; 21: 2385-2398.
2. Zhu S, Chen Z, Wang R, Tan Y, Ge M, Sun Y*,* et al*.* MLL is required for miRNA-mediated translational repression. Cell Discov. 2019; (doi:10.1038/s41421-019-0111-0).

3. Doench J.G., Petersen C.P. & Sharp P.A. siRNAs can function as miRNAs. Genes Dev. 2003; 17: 438-442.

**Supplementary Figure and Figure Legends**

**
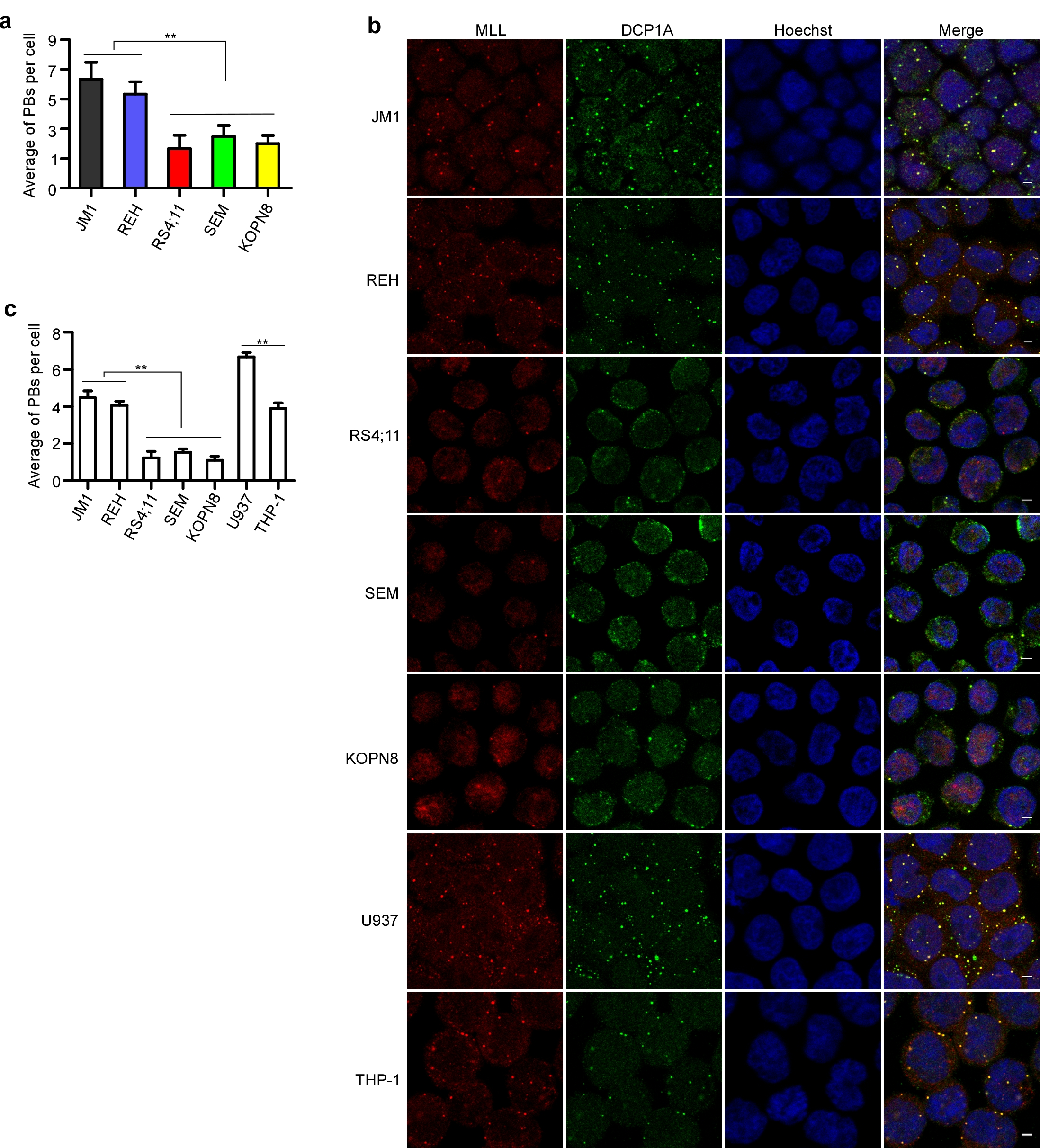
**

**Supplementary Figure S1.** **P-body formation was affected in MLL leukemic cells.** **a** Detectable DDX6-associated P-bodies in MLL leukemia cells were quantified for all cells in the field of view (126x magnification) and data from at least 3 random fields were collected and analyzed using Image J. JM1 and REH cells harbor wild-type *MLL* gene, RS4;11 and SEM cells harbor *MLL-AF4* gene, KOPN8 cells harbor *MLL-ENL* gene. **b** JM1, REH, RS4;11, SEM, KOPN8, U937 and THP-1 cell lines were probed with antibodies to DCP1A for immunofluorescence assay. JM1, REH and U937 cells harbor wild-type *MLL* gene, RS4;11 and SEM cells harbor *MLL-AF4* gene, KOPN8 cells harbor *MLL-ENL* gene, THP-1 cells harbor *MLL-AF9* gene. Scale bar, 5 μm. **c** Detectable DCP1A-associated P-bodies in MLL leukemia cells were quantified for all cells in the field of view (126x magnification) and data from at least 3 random fields were collected and analyzed using Image J. * for *P*<0.05, ** for *P*<0.01. Data represent mean and s.e.m of three independent experiments.

**
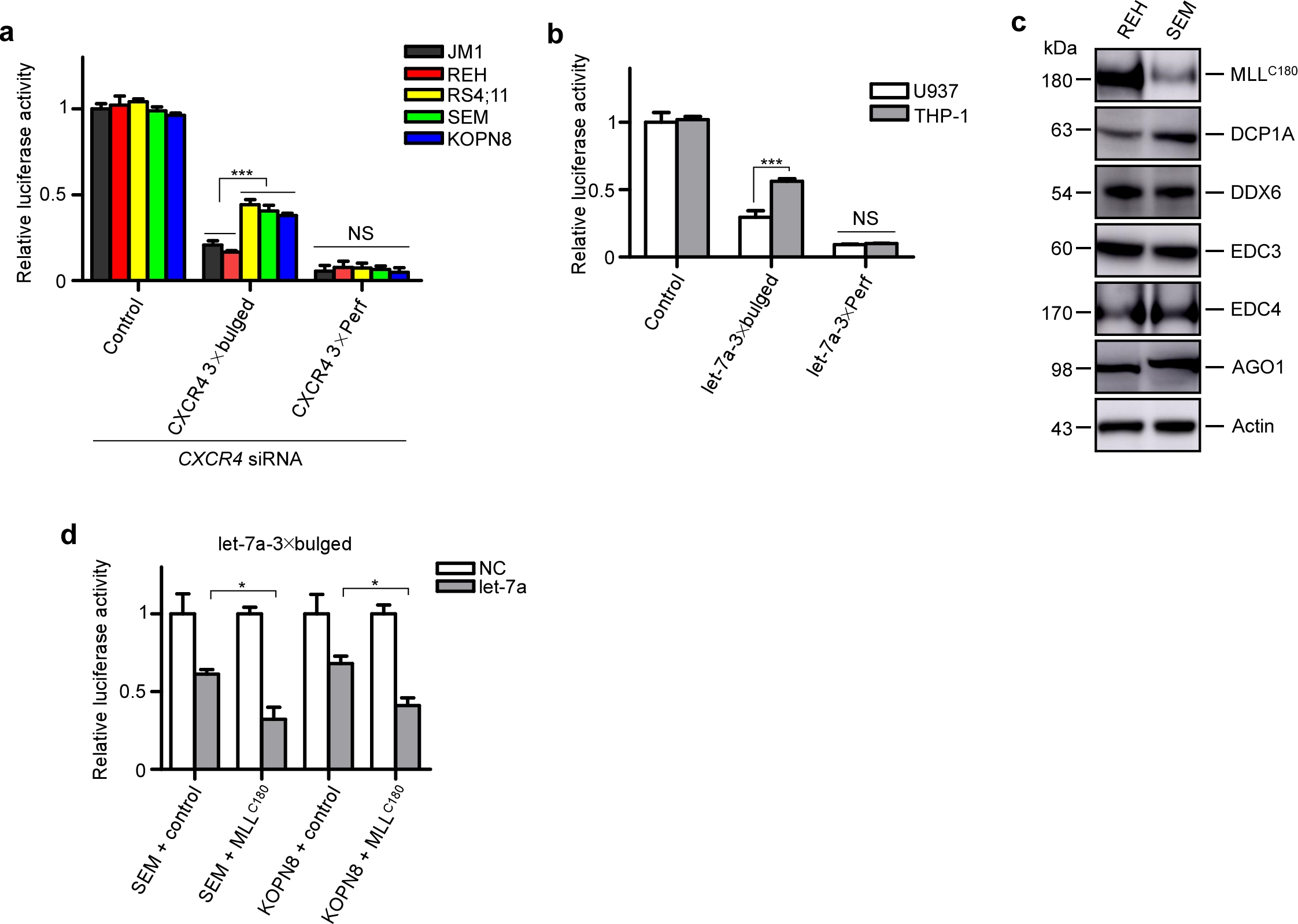
**

**Supplementary Figure S2. MLL-fusion leukemic cells presented an impaired miRNA-mediated translational repression.** **a** *CXCR4* reporter assays were performed as described in Fig. 1a ii. The reporter activity was normalized to JM1 cells co-transfected with empty reporter vector and siCXCR4. **b** miRNA reporter assays were performed as described in Fig. 1a ii. The reporter activity was measured and normalized to U937 cells transfected with empty reporter vector. **c** The expression level of P-body proteins in REH and SEM. Total lysates of REH and SEM cells were subjected to Western blot with indicated antibodies. **d** SEM and KOPN8 cells were transduced with empty vector or *MLL^C180^*, to determine its role in the dysregulated *let-7a* mediated gene silencing. Experiments were performed as described in Fig. 1a ii. The ratio of luciferase activity was measured and normalized to the value of the cells transfected with the NC, respectively. * for *P*<0.05, ** for *P*<0.01, *** for *P*<0.001. NS, no significant difference. Data represent mean and s.e.m of three independent experiments.


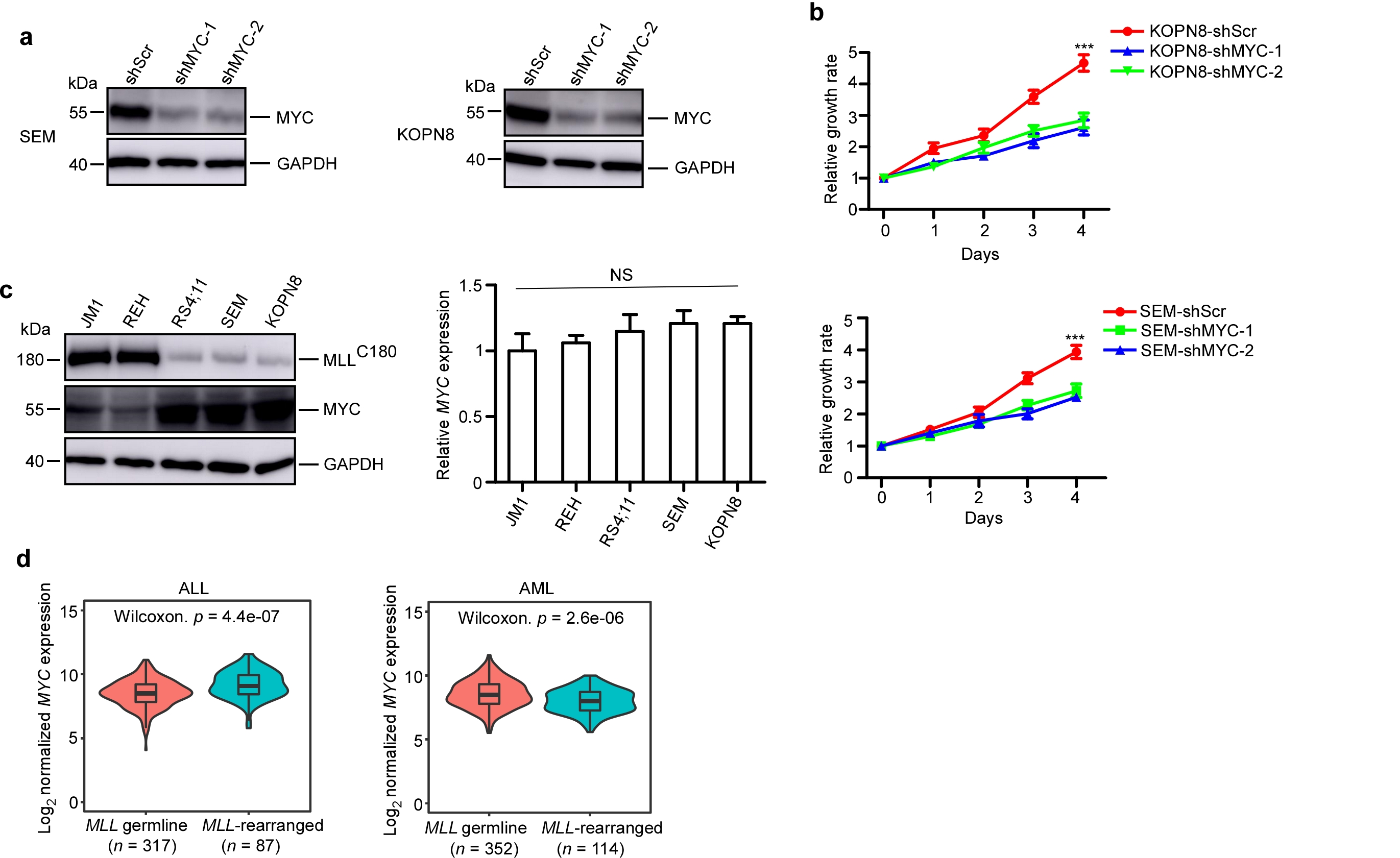


**Supplementary Figure S3. MLL leukemic cells were generally dependent on high levels of MYC.** **a** Western blot assays were performed to evaluate the *MYC* knockdown by targeting two independent sequences in SEM and KOPN8 cell line. GAPDH serves as a loading control. **b** The proliferation of SEM and KOPN8 cells upon *MYC* depletion were quantified by MTS assay. **c** MYC protein and mRNA levels in MLL leukemia cells were analyzed by western blot (left) and qRT-PCR (right), respectively. *MYC* mRNA level was quantified relative to JM1. *GAPDH* was used as control in qRT-PCR experiments. **d** *MYC* expression levels in primary ALL (left) and AML (right) patient samples were shown by analyzing the publicly available microarray datasets. Samples were dichotomized according to the *MLL* status. Wilcoxon rank-sum tests were performed. Sample size and *P*-values were presented. * for *P*<0.05, ** for *P*<0.01, *** for *P*<0.001. NS, no significant difference. Data represent mean and s.e.m of three independent experiments.


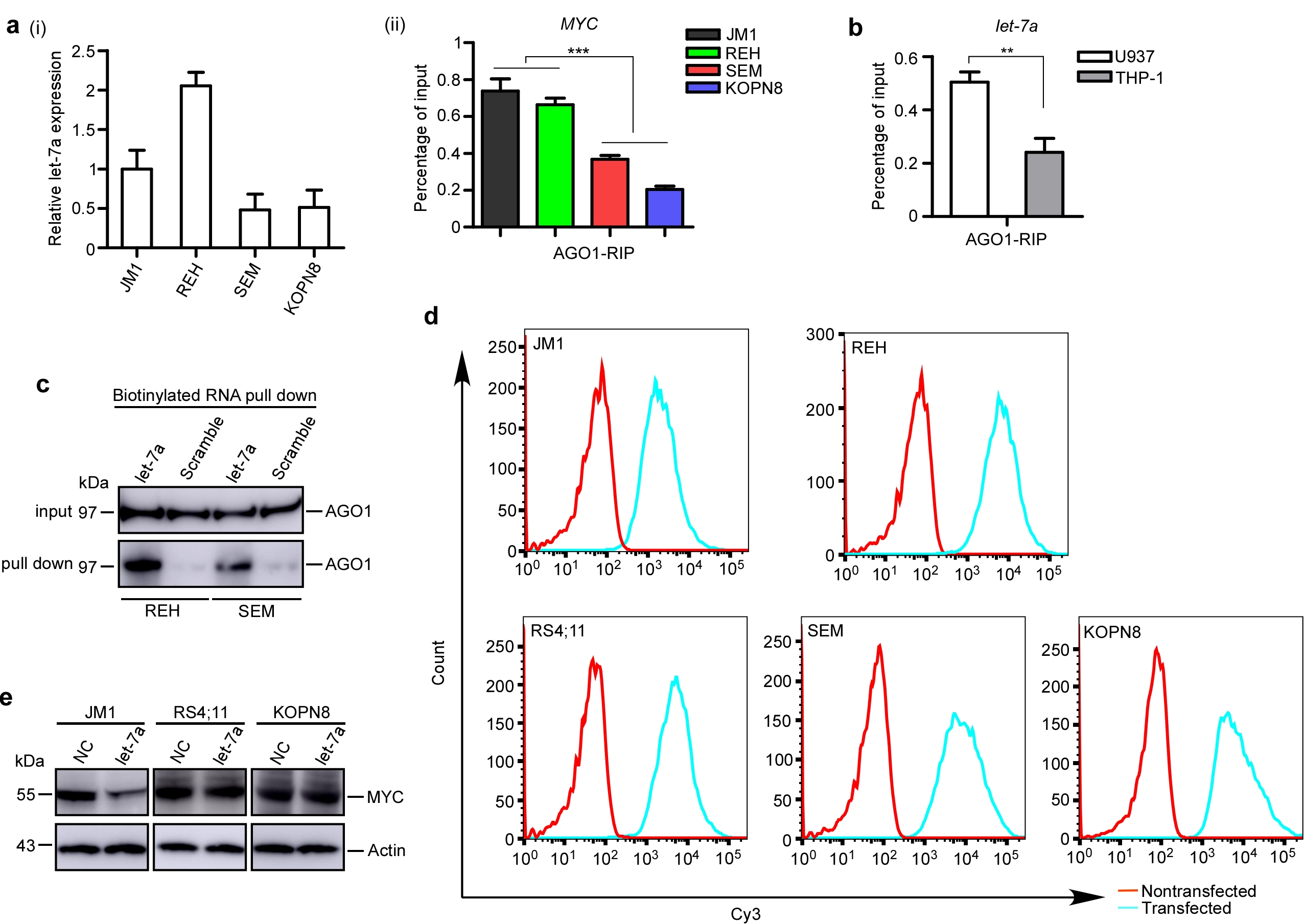


**Supplementary Figure S4. Translational repression of *MYC* was reduced in MLL leukemic cells.** **a (i)** *let-7a* levels in MLL leukemia cells were analyzed by qRT-PCR using specific primers. Expression levels were quantified relative to JM1. *RNU6B* was used as control in qRT-PCR experiments. **(ii)** Extracts of JM1, REH, SEM and KOPN8 cells were subjected to anti-AGO1 RIP assays. The pull-downed RNAs were analyzed by qRT-PCR using primers for *MYC*. **b** Extracts of U937 and THP-1 cells were subjected to anti-AGO1 RIP assays. The pull-downed RNAs were analyzed by qRT-PCR using primers for *let-7a*. **c** REH and SEM cellular lysates were subjected to Biotinylated-let-7a RNA pull down assay. Then let-7a-immunoprecipitated AGO1 proteins were subjected to the Western blot analysis. Scrambled miRNAs were used as negative control. **d** Representative flow cytometry analyses of *MLL*-wild type and *MLL*-rearranged cell lines transfected with let-7a. **e** JM1, RS4;11 and KOPN8 cells were transfected with NC and let-7a. Proteins were detected by western blot assays with anti-MYC antibody at 24h post-transfection. * for *P*<0.05, ** for *P*<0.01, *** for *P*<0.001. Data represent mean and s.e.m of three independent experiments.

**
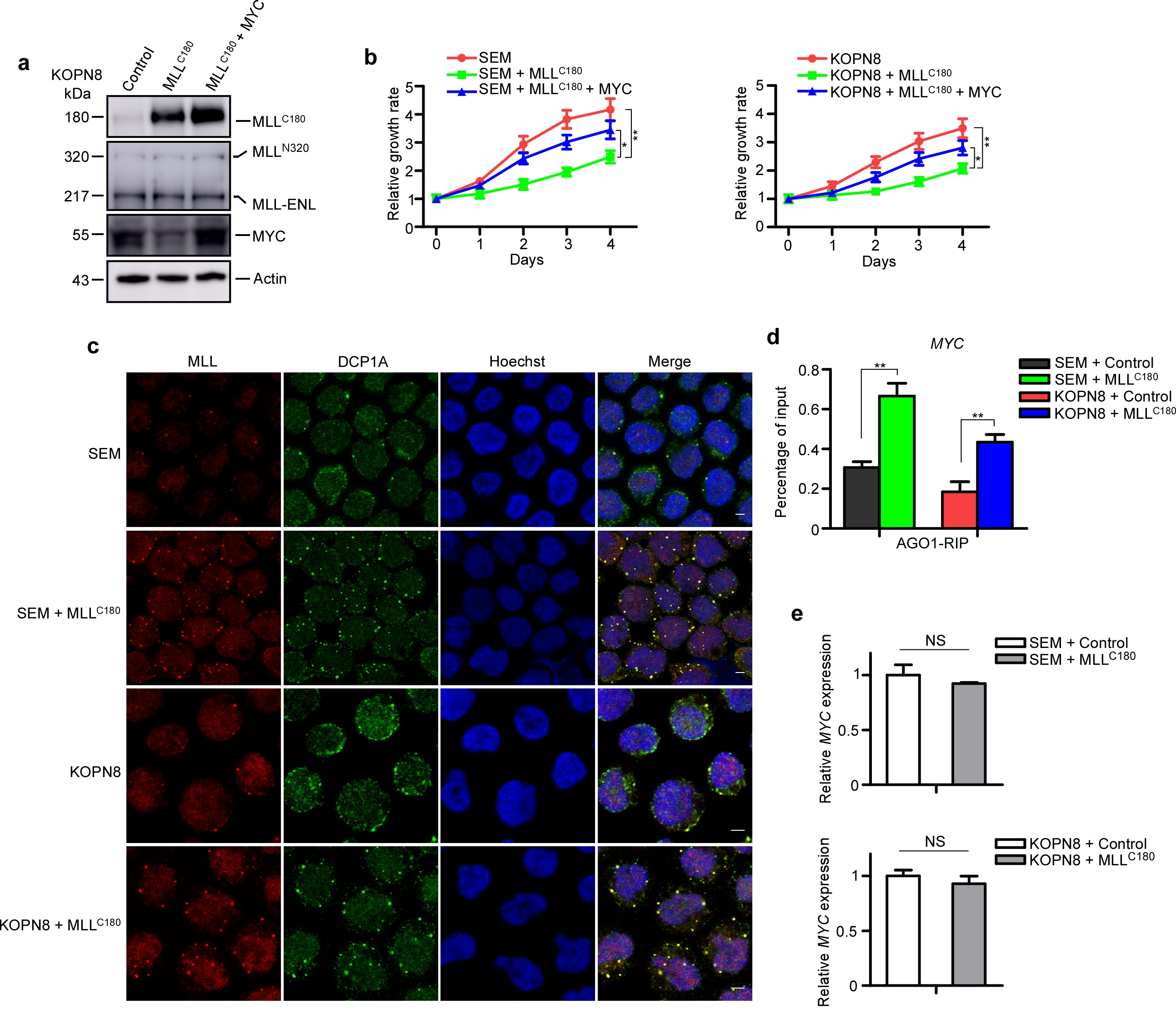
**

**Supplementary Figure S5. The reduction of MLL^C180^ played a causal role in the miRNA functional deficiency in MLL leukemic cells. a** KOPN8 cells transduced with *MLL^C180^*, together with or without *MYC*, were subjected to Western blot assays. Antibodies were used as indicated. MLL-ENL fusion proteins were detected using antibody which specifically recognizing the amino terminus of MLL. **b** MTS assays displaying the effect of *MYC* on restoring cell proliferation in *MLL^C180^*-transduced SEM and KOPN8 cells. **c** SEM and KOPN8 cells transduced with or without *MLL^C180^* were probed with antibodies to DCP1A for immunofluorescence assays. Scale bar, 5 μm. **d** SEM and KOPN8 cells transduced with or without *MLL^C180^* were subjected to anti-AGO1 RIP assays. Pull-down RNAs were analyzed by qRT-PCR using primers for *MYC*. **e** SEM (upper) and KOPN8 (lower) cells were transduced with or without *MLL^C180^*, cells were then collected for the qRT-PCR assays to analyze the *MYC* mRNA expression levels. Values were quantified relative to the cells transduced with Control. *GAPDH* was used as internal control in qRT-PCR experiments. * for *P*<0.05, ** for *P*<0.01. NS, no significant difference. Data represent mean and s.e.m of three independent experiments.


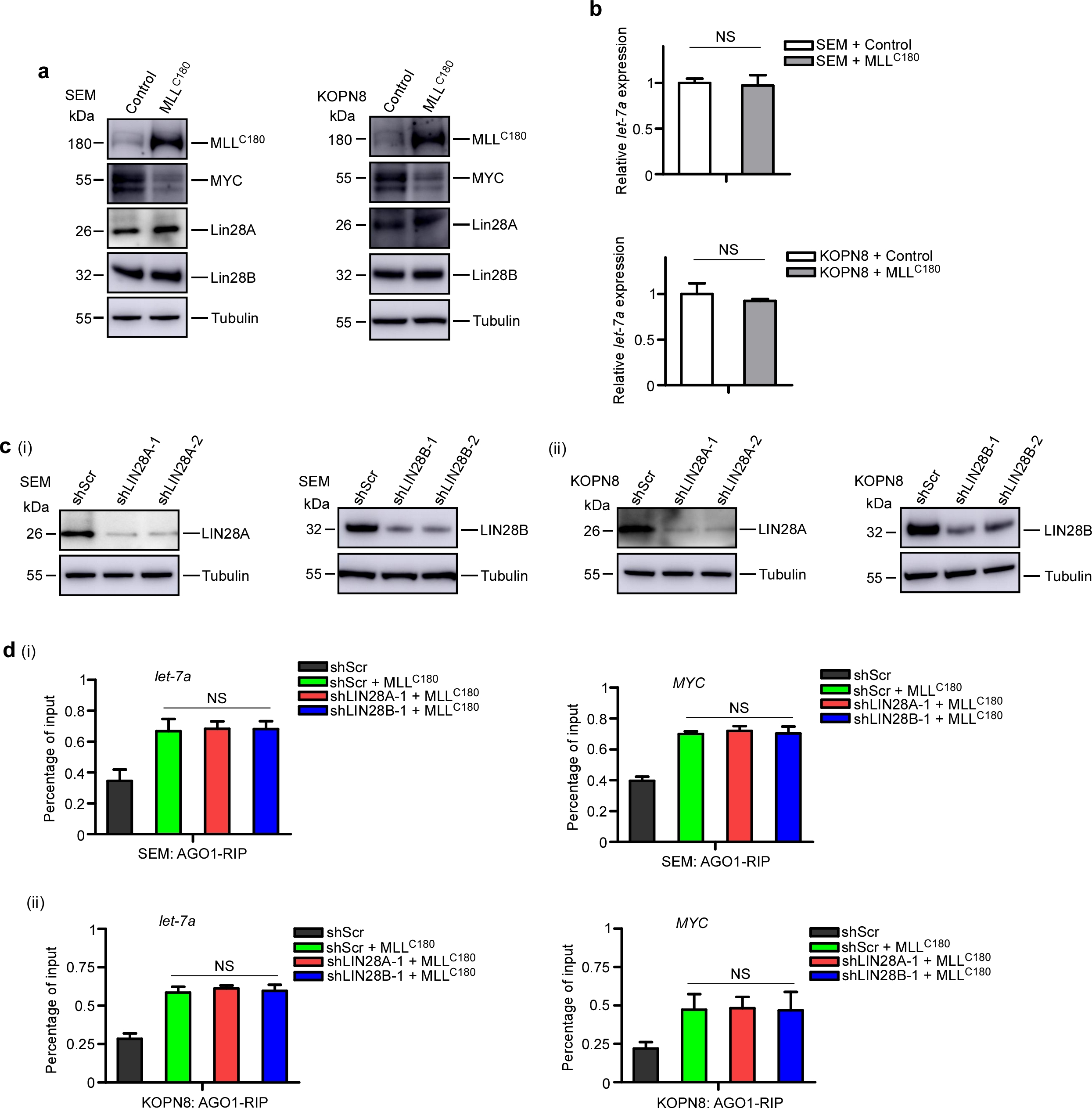


**Supplementary Figure S6. The modulation of MLL^C180^ in controlling translational repression of *MYC* by *let-7a* was not dependent on LIN28.** **a** SEM and KOPN8 cells transduced with Control or *MLL^C180^* were collected for the Western blot assays. Antibodies were used as indicated. **b** SEM and KOPN8 cells transduced with Control or *MLL^C180^* were subjected to the qRT-PCR assays. *let-7a* levels were analyzed using specific primers and values were quantified relative to the cells transduced with Control. *RNU6B* was used as internal control in qRT-PCR experiments. **c** Western blot assays were performed to evaluate the *LIN28A* and *LIN28B* knockdown by targeting two independent sequences in SEM (i) and KOPN8 (ii) cell lines. Tubulin serves as a loading control. **d** The binding of *let-7a* and *MYC* mRNA to the AGO1 in SEM (i) and KOPN8 (ii) cells upon *LIN28A* and *LIN28B* depletion were analyzed by anti-AGO1 RIP assays. Pull-down RNAs were analyzed by qRT-PCR using specific primers for *let-7a* and *MYC*, respectively. NS, no significant difference. Data represent mean and s.e.m of three independent experiments.


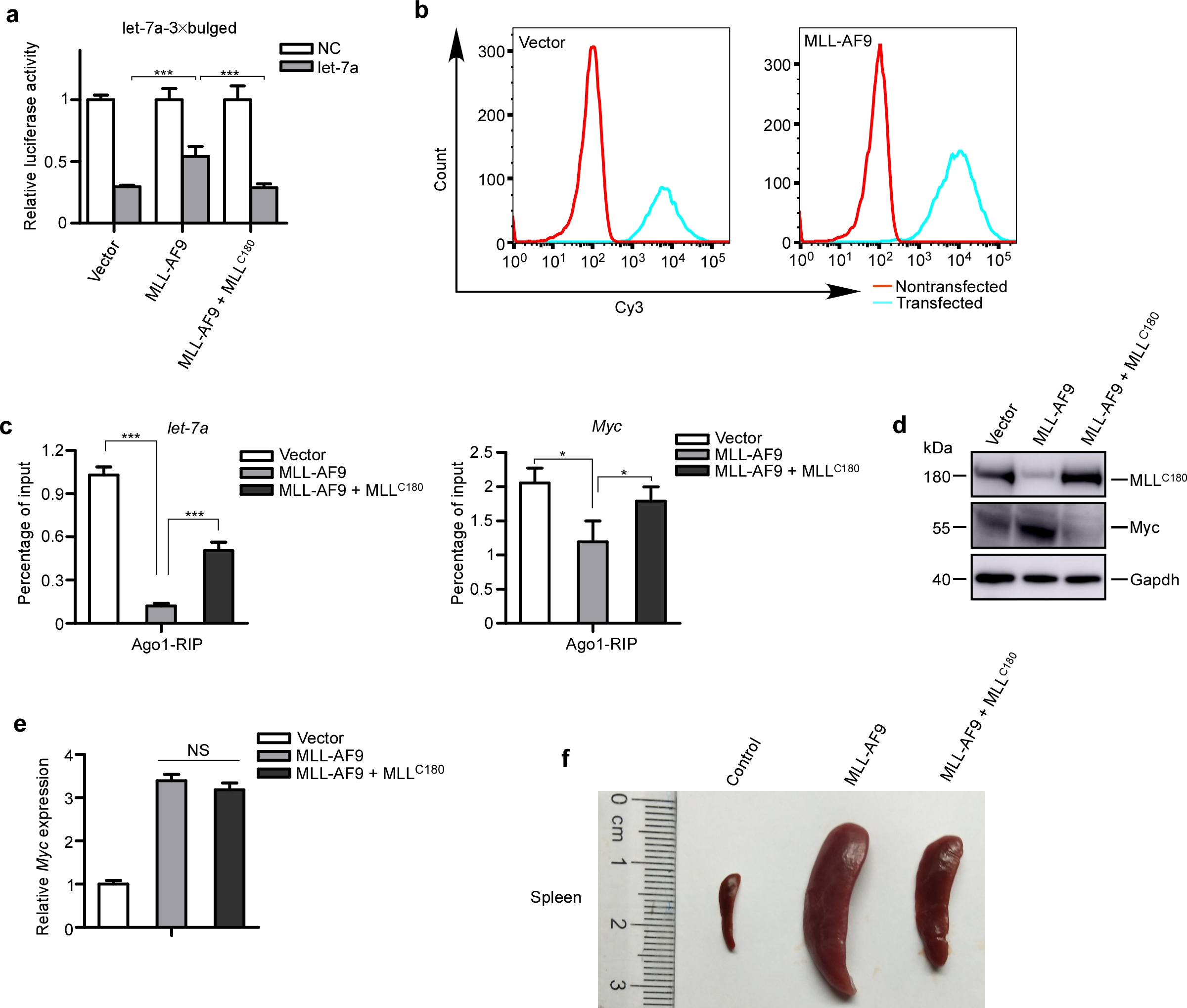


**Supplementary Figure S7.** **Impaired miRNA-mediated translational repression in MLL-fusion leukemic cells contributes to MYC dependent survival. a** Mouse bone marrow cells transduced with empty vector (control), *MLL-AF9*, together with or without *MLL^C180^* (MLL-AF9 + MLL^C180^) were collected for the luciferase reporter assays. Cells transfected with Agomir-negative control (NC) or Agomir-let-7a mimic (let-7a) were subjected to dual luciferase reporter assays. The ratio of luciferase activity was measured and normalized to the value of the cells transfected with the NC, respectively. **b** Representative flow cytometry analyses of *MLL*-*AF9*-transduced primary mouse bone marrow progenitor cells and control cells transfected with let-7a. **c** Mouse bone marrow cells transduced with empty vector (control), *MLL-AF9*, together with or without *MLL^C180^* (MLL-AF9 + MLL^C180^) were collected, for the anti-Ago1 RIP experiments. Pull-downed RNAs were analyzed by qRT-PCR using specific primers for *let-7a* (left) and *Myc* (right). **d** Mouse bone marrow cells transduced with empty vector (control), *MLL-AF9*, together with or without *MLL^C180^* (MLL-AF9 + MLL^C180^) were collected for the western blot assays using the indicated antibodies. **e** Mouse bone marrow cells transduced with empty vector (control), *MLL-AF9*, together with or without *MLL^C180^* (MLL-AF9 + MLL^C180^) were collected for the qRT-PCR assays. *Myc* mRNA levels were analyzed using specific primers and values were quantified relative to the cells transduced with Vector. *Gapdh* was used as internal control in qRT-PCR experiments. **f** *MLL-AF9* primary cells transduced with or without *MLL^C180^* were transplanted into mice to induce leukemia (*n* = 5 per each group). Representative photograph of spleens was showed. The size of the spleens can be seen using the scale of the ruler, each grid represents 1 mm. * for *P*<0.05, ** for *P*<0.01, *** for *P*<0.001. NS, no significant difference. Data represent mean and s.e.m of three independent experiments.


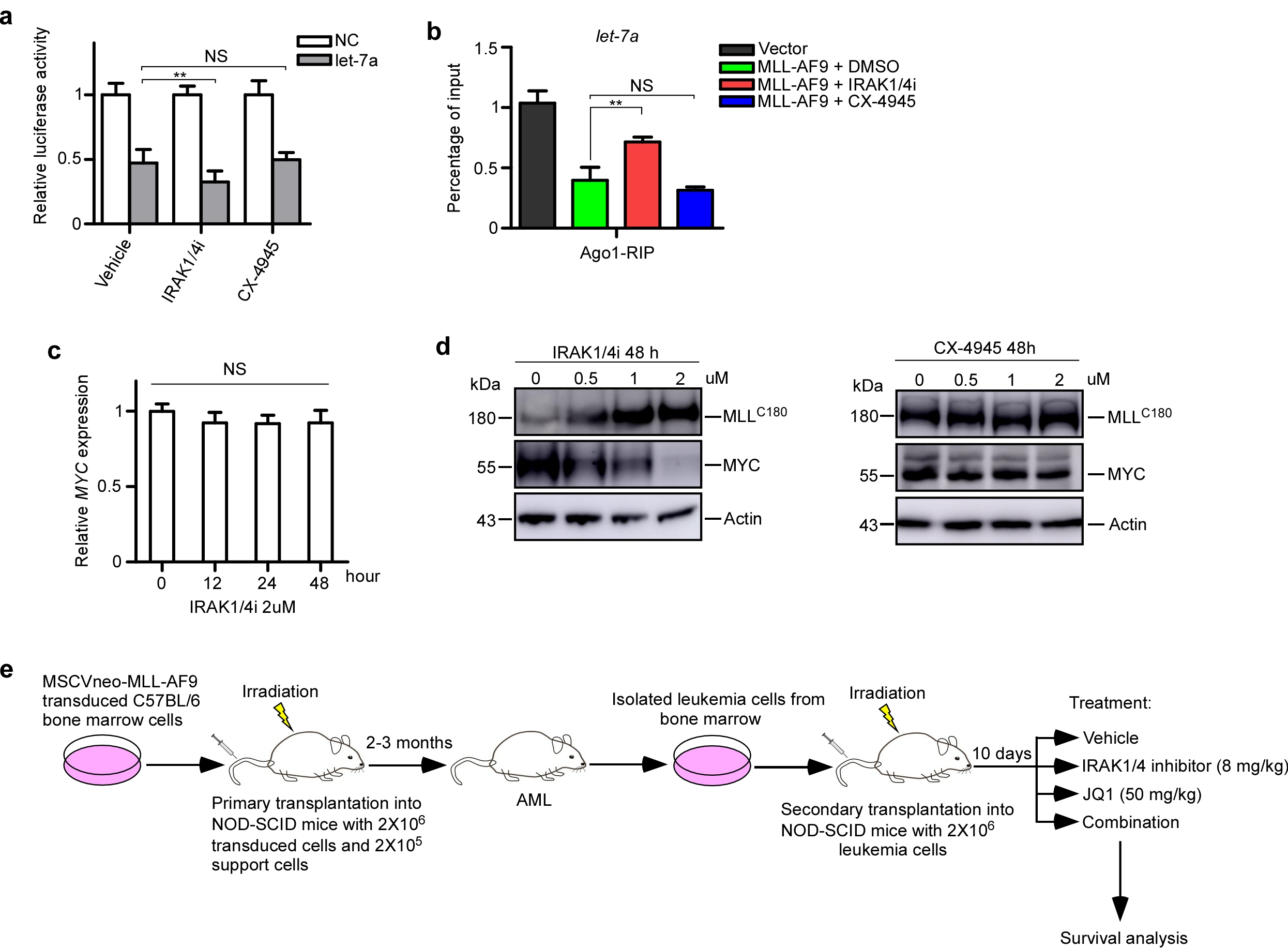


**Supplementary Figure S8.** **IRAK1/4i could rescue the function of *let-7a* and decrease the expression of MYC via restoration of MLL protein levels. a** *MLL-AF9* primary leukemia cells were treated with vehicle (control), IRAK1/4 inhibitor (2 μM) and CKII inhibitor CX-4945 (2 μM) for 24 h. Then, cells were transfected with Agomir-negative control (NC) or Agomir-let-7a mimic (let-7a) and subjected to dual luciferase reporter assays. The ratio of luciferase activity was measured and normalized to the value of the cells transfected with the NC. **b** Mouse bone marrow cells transduced with empty vector (control) or *MLL-AF9*, were treated with DMSO (control), IRAK1/4 inhibitor (2 μM) or CX-4945 (2 μM) for 48 h. Then cells were collected for the Anti-Ago1 RIP assays. Pull-down RNAs were analyzed by qRT-PCR using primers for *let-7a*. **c** qRT-PCR analyses of the time-dependent effects of IRAK1/4 inhibitor treatment on *MYC* mRNA expression in *MLL-AF9* primary leukemia cells. *MYC* mRNA levels were analyzed using specific primers and values were quantified relative to the untreated cells. *GAPDH* was used as internal control in qRT-PCR experiments. **d** *MLL-AF9* primary leukemia cells were treated with the indicated doses of IRAK1/4 inhibitor (left) or CX-4945 (right) for 48 h. Western blot assays were performed using the indicated antibodies. **e** Schematic of the development of secondary murine *MLL-AF9* leukemia and IRAK1/4 inhibitor and JQ1 treatment strategies in a *MLL-AF9* mouse model (*n* = 5 per each group). Drug treatments were initiated at day 10 after leukemia cells transplantation. * for *P*<0.05, ** for *P*<0.01. NS, no significant difference. Data represent mean and s.e.m of three independent experiments.
